# Supplementary material for: Genome-scale rates of evolutionary change in bacteria
Source: Microb Genom. 2016 Nov 30;2(11):e000094. doi: 10.1099/mgen.0.000094 (PMC5320706; doi:10.1099/mgen.0.000094)
Supplement: Supplementary File 1 [file mgen-02-94-s001.pdf]

Supplementary material

Figure S1. Regression of root-to-tip distance (expected substitutions per site) as a function of sampling time for an *Acinetobacter baumanii* GC2 data set. The points in red correspond to the phylogenetic tree using all SNPs in the genome, and therefore includes recombining sites. The points in blue were obtained after filtering sites with evidence of recombination using Gubbins. The lines represent the linear regression for each set of points. The nucleotide substitution rate estimates for the SNP data and whole genomes are shown for both sets of points. Importantly, that the regression line in red has a negative slope incorrectly suggests a lack of temporal structure and an over-dispersed molecular clock, whereas that in blue correctly demonstrates that the data have considerable temporal structure and reasonable clock-like behavior.

Figure S2. Estimated genomic substitution rates in human-associated bacterial pathogens and tip-date randomisations. Points correspond to the mean estimate and the error bars are the 95% HPD values. Data points in black are for the estimates obtained with the correct sampling times, while those in orange were obtained using the tip-date randomisations.

Figure S3. Heatmap of statistical support for different combinations of molecular clock and demographic models, estimated using marginal likelihood. Rows correspond to bacterial data sets and are sorted in descending order according to their Bayesian mean genomic rate estimate, as shown. The columns represent the different combinations of molecular clock and demographic models; cell colours indicate the ranking of statistical support for each data set; from red (highest marginal likelihood) to light yellow (lowest marginal likelihood).

Figure S4. Residual plots for rate as a function of sampling time according to the double exponential decay function $y=-5.93/x^{-0.17}$, where y and x correspond to the substitution rate and sampling time in $\log_{10}$ scale. (A) shows the residuals as a function of the fitted substitution rate, where each point corresponds to a bacterial data set. (B) is the probability density of the residuals.

Table S1. Information on the bacterial data sets used in this study, including parameter estimates and the corresponding references.

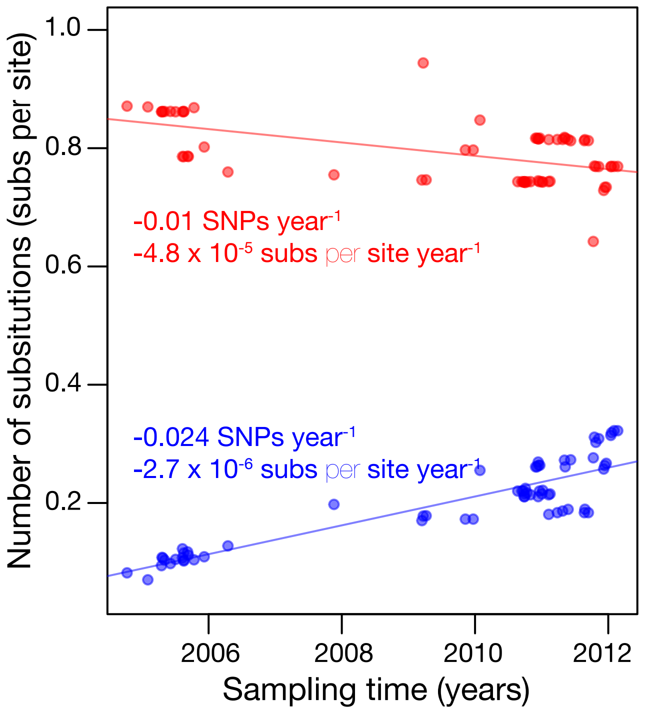

Number of subsitutions (subs per site)
1.0
0.8
0.6
0.4
0.2
-0.01 SNPs year-1
-4.8 x 10-5 subs per site year-1
-0.024 SNPs year-1
-2.7 x 10-6 subs per site year-1
2006
2008
2010
2012
Sampling time (years)

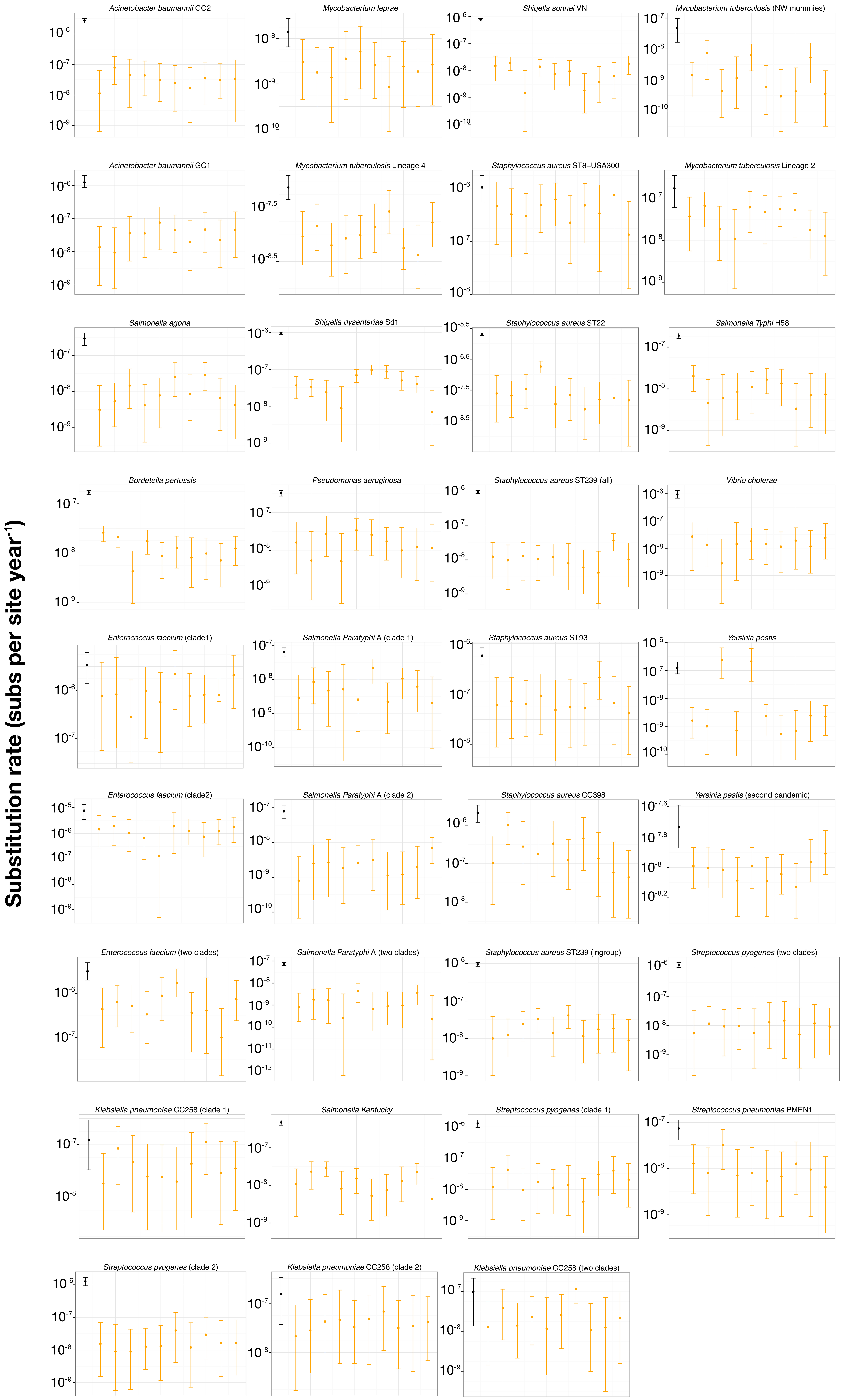
Substitution rate (subs per site year^{-1})
Acinetobacter baumannii GC2
Mycobacterium leprae
Shigella sonnei VN
Mycobacterium tuberculosis (NW mummies)
Acinetobacter baumannii GC1
Mycobacterium tuberculosis Lineage 4
Staphylococcus aureus ST8–USA300
Mycobacterium tuberculosis Lineage 2
Salmonella agona
Shigella dysenteriae Sd1
Staphylococcus aureus ST22
Salmonella Typhi H58
Bordetella pertussis
Pseudomonas aeruginosa
Staphylococcus aureus ST239 (all)
Vibrio cholerae
Enterococcus faecium (clade1)
Salmonella Paratyphi A (clade 1)
Staphylococcus aureus ST93
Yersinia pestis
Enterococcus faecium (clade2)
Salmonella Paratyphi A (clade 2)
Staphylococcus aureus CC398
Yersinia pestis (second pandemic)
Enterococcus faecium (two clades)
Salmonella Paratyphi A (two clades)
Staphylococcus aureus ST239 (ingroup)
Streptococcus pyogenes (two clades)
Klebsiella pneumoniae CC258 (clade 1)
Salmonella Kentucky
Streptococcus pyogenes (clade 1)
Streptococcus pneumoniae PMEN1
Streptococcus pyogenes (clade 2)
Klebsiella pneumoniae CC258 (clade 2)
Klebsiella pneumoniae CC258 (two clades)

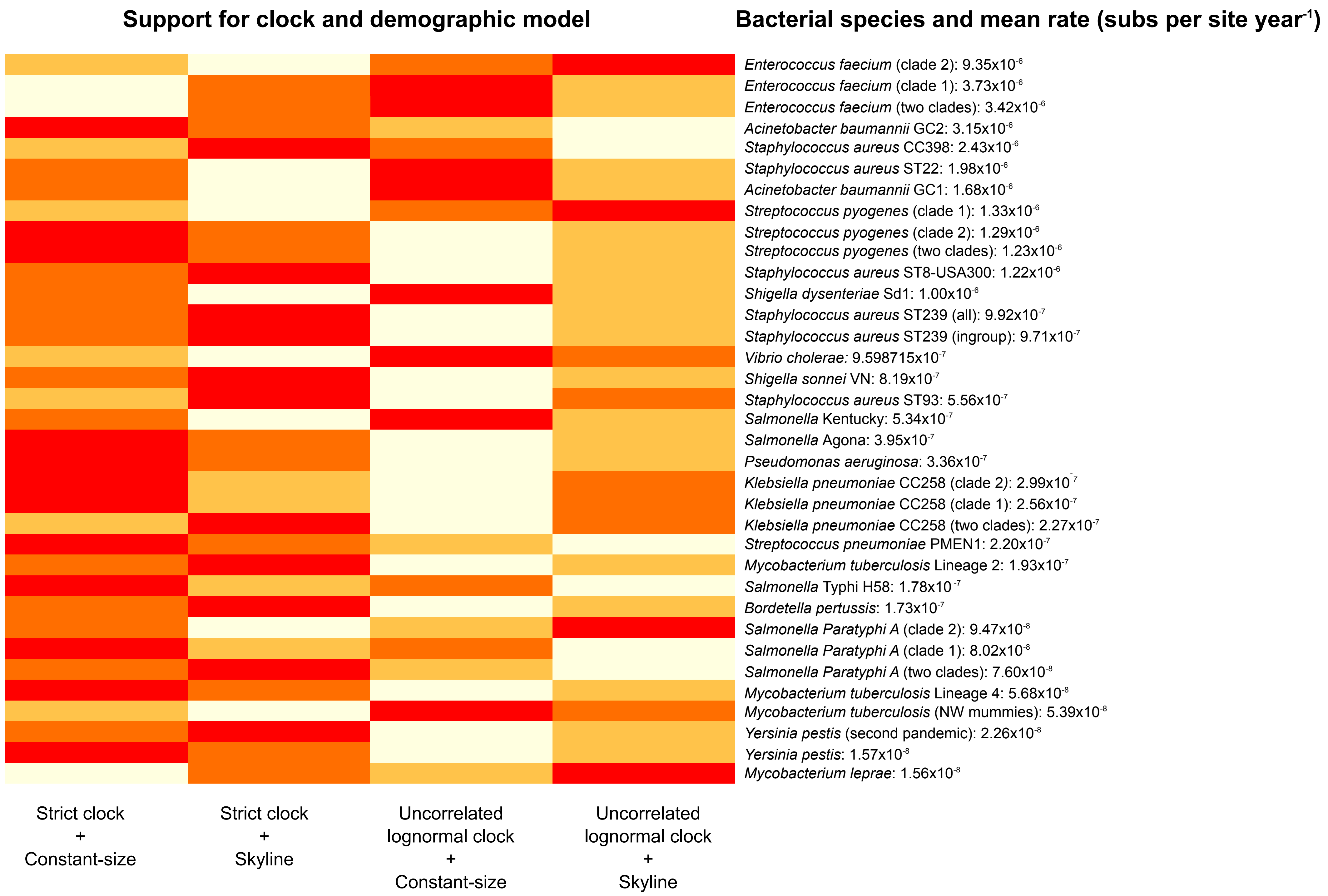
Support for clock and demographic model
Bacterial species and mean rate (subs per site year^{-1})
Enterococcus faecium (clade 2): 9.35x10^{-6}
Enterococcus faecium (clade 1): 3.73x10^{-6}
Enterococcus faecium (two clades): 3.42x10^{-6}
Acinetobacter baumannii GC2: 3.15x10^{-6}
Staphylococcus aureus CC398: 2.43x10^{-6}
Staphylococcus aureus ST22: 1.98x10^{-6}
Acinetobacter baumannii GC1: 1.68x10^{-6}
Streptococcus pyogenes (clade 1): 1.33x10^{-6}
Streptococcus pyogenes (clade 2): 1.29x10^{-6}
Streptococcus pyogenes (two clades): 1.23x10^{-6}
Staphylococcus aureus ST8-USA300: 1.22x10^{-6}
Shigella dysenteriae Sd1: 1.00x10^{-6}
Staphylococcus aureus ST239 (all): 9.92x10^{-7}
Staphylococcus aureus ST239 (ingroup): 9.71x10^{-7}
Vibrio cholerae: 9.598715x10^{-7}
Shigella sonnei VN: 8.19x10^{-7}
Staphylococcus aureus ST93: 5.56x10^{-7}
Salmonella Kentucky: 5.34x10^{-7}
Salmonella Agona: 3.95x10^{-7}
Pseudomonas aeruginosa: 3.36x10^{-7}
Klebsiella pneumoniae CC258 (clade 2): 2.99x10^{-7}
Klebsiella pneumoniae CC258 (clade 1): 2.56x10^{-7}
Klebsiella pneumoniae CC258 (two clades): 2.27x10^{-7}
Streptococcus pneumoniae PMEN1: 2.20x10^{-7}
Mycobacterium tuberculosis Lineage 2: 1.93x10^{-7}
Salmonella Typhi H58: 1.78x10^{-7}
Bordetella pertussis: 1.73x10^{-7}
Salmonella Paratyphi A (clade 2): 9.47x10^{-8}
Salmonella Paratyphi A (clade 1): 8.02x10^{-8}
Salmonella Paratyphi A (two clades): 7.60x10^{-8}
Mycobacterium tuberculosis Lineage 4: 5.68x10^{-8}
Mycobacterium tuberculosis (NW mummies): 5.39x10^{-8}
Yersinia pestis (second pandemic): 2.26x10^{-8}
Yersinia pestis: 1.57x10^{-8}
Mycobacterium leprae: 1.56x10^{-8}
Strict clock + Constant-size
Strict clock + Skyline
Uncorrelated lognormal clock + Constant-size
Uncorrelated lognormal clock + Skyline

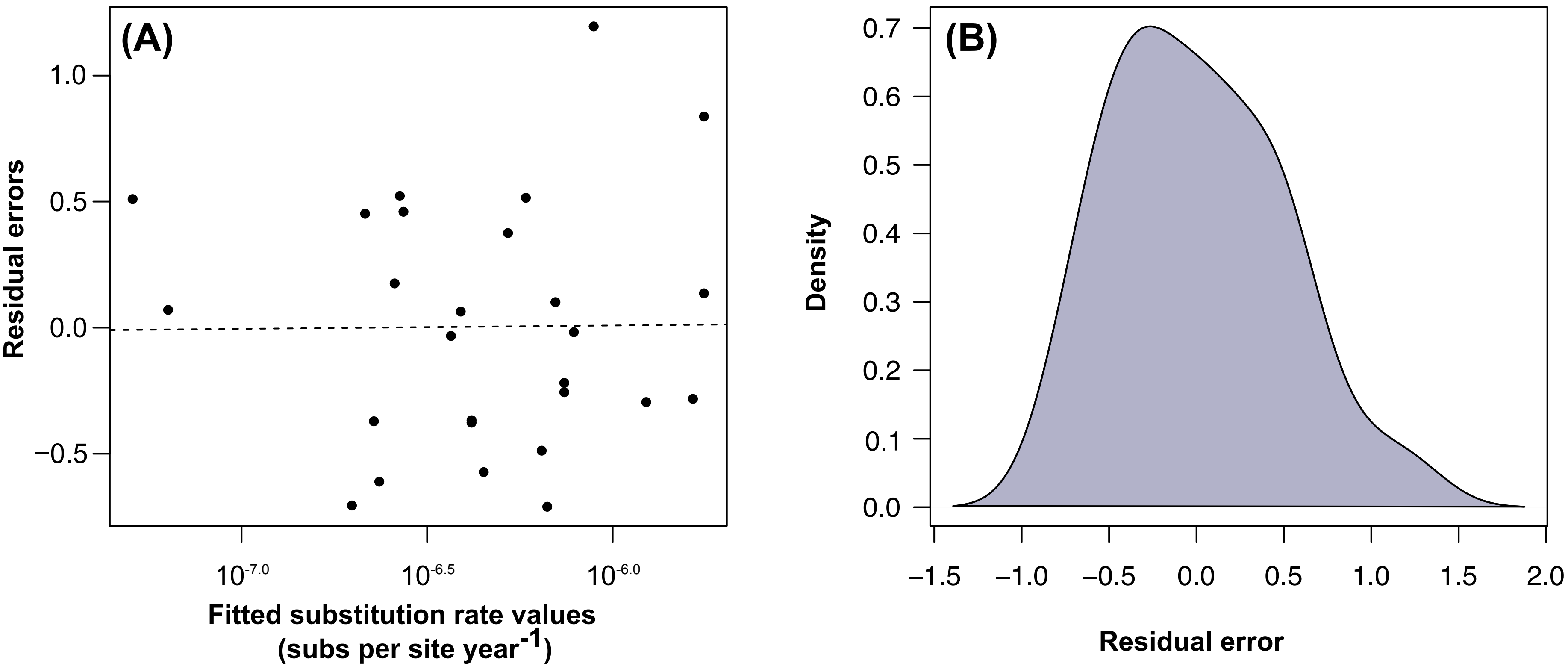

(A)
Residual errors
1.0
0.5
0.0
−0.5
$10^{-7.0}$
$10^{-6.5}$
$10^{-6.0}$
Fitted substitution rate values
(subs per site year^{-1})
(B)
Density
0.7
0.6
0.5
0.4
0.3
0.2
0.1
0.0
−1.5
−1.0
−0.5
0.0
0.5
1.0
1.5
2.0
Residual error

Number of recombinant	RDP methods	Species	Model	CR1	CR2	Regression rate (subs per site year^{-1})	Beast genomic rate (subs per site year^{-1})	Sampling time (years)
0	None	*Mycobacterium leprae*	strict/constant	0.8	0.4	4.63 $\times10^{-9}$	1.56216 $\times10^{-8}$	1993
0	None	*Yersinia pestis*	ucld/constant	1	0.8	6.88 $\times10^{-9}$	1.57264 $\times10^{-8}$	1506
0	None	*Yersinia pestis*	ucld/constant	1	0.5	-6.27 $\times10^{-9}$	2.26097 $\times10^{-8}$	657
0	None	*Mycobacterium tuberculosis*	ucld/constant	1	0.9	6.30 $\times10^{-8}$	5.67521 $\times10^{-8}$	13.17
46	RGBMST	*Neisseria meningitidis*	ucld/constant	0.7	0	3.34 $\times10^{-7}$	6.04966 $\times10^{-8}$	59
0	None	*Salmonella enterica*	ucld/constant	1	1	5.43 $\times10^{-8}$	7.60404 $\times10^{-8}$	84
0	None	*Salmonella enterica*	ucld/constant	1	1	4.77 $\times10^{-8}$	8.01648 $\times10^{-8}$	60
0	None	*Salmonella enterica*	strict/constant	1	1	6.83 $\times10^{-8}$	9.47397 $\times10^{-8}$	58
0	None	*Bordetella pertussis*	ucld/constant	1	1	2.51 $\times10^{-7}$	1.72569 $\times10^{-7}$	63
0	None	*Salmonella enterica*	ucld/skyline	1	1	1.26 $\times10^{-7}$	1.78159 $\times10^{-7}$	21
0	None	*Mycobacterium tuberculosis*	ucld/constant	1	0.3	-7.59 $\times10^{-9}$	1.92695 $\times10^{-7}$	16
0	None	*Streptococcus pneumoniae*	ucld/skyline	1	0.9	1.61 $\times10^{-7}$	2.20099 $\times10^{-7}$	24
0	None	*Pseudomonas aeruginosa*	ucld/constant	1	1	2.59 $\times10^{-7}$	3.36228 $\times10^{-7}$	35
0	None	*Salmonella enterica*	ucld/constant	1	1	1.13 $\times10^{-7}$	3.95293 $\times10^{-7}$	38
0	None	*Salmonella enterica*	ucld/constant	1	1	2.89 $\times10^{-7}$	5.34566 $\times10^{-7}$	77
0	None	*Staphylococcus aureus*	strict/skyline	1	0.9	2.63 $\times10^{-7}$	5.55592 $\times10^{-7}$	17
0	None	*Klebsiella pneumoniae*	strict/skyline	8	0	1.27 $\times10^{-7}$	2.26961 $\times10^{-7}$	7
0	None	*Klebsiella pneumoniae*	strict/skyline	6	0.8	1.25 $\times10^{-7}$	2.56136 $\times10^{-7}$	7
0	None	*Shigella dysenteriae*	ucld/constant	1	1	8.02 $\times10^{-7}$	1.00633 $\times10^{-6}$	96
0	None	*Shigella sonnei*	ucld/constant	1	1	6.33 $\times10^{-7}$	8.19116 $\times10^{-7}$	15

0	None	*Klebsiella pneumoniae*	strict/skyline	8	0	3.05×10^{-7}	2.98841×10^{-7}	6
0	None	*Vibrio cholerae*	strict/skyline	1	1	9.80×10^{-7}	9.59872×10^{-7}	73
0	None	*Staphylococcus aureus*	ucld/constant	1	1	8.05×10^{-7}	9.70923×10^{-7}	32
0	None	*Staphylococcus aureus*	ucld/constant	1	1	8.05×10^{-7}	9.92258×10^{-7}	32
0	None	*Staphylococcus aureus*	ucld/constant	0.4	0	8.20×10^{-7}	1.22035×10^{-6}	2.15
0	None	*Streptococcus pyogenes*	ucld/constant	1	1	9.60×10^{-7}	1.22659×10^{-6}	16
0	None	*Streptococcus pyogenes*	ucld/constant	1	1	9.88×10^{-7}	1.28798×10^{-6}	7
0	None	*Streptococcus pyogenes*	strict/constant	1	1	1.05×10^{-6}	1.33584×10^{-6}	16
0	None	*Acinetobacter baumannii*	ucld/constant	1	1	1.38×10^{-6}	1.68093×10^{-6}	29
5	RGBMST	*Staphylococcus aureus*	ucld/constant	1	1	1.57×10^{-6}	1.98×10^{-6}	18.72
4	RGBMST	*Enterococcus faecium*	ucld/constant	0.6	0	2.33×10^{-6}	3.73×10^{-6}	7
0	None	*Acinetobacter baumannii*	ucld/skyline	1	1	2.53×10^{-6}	3.15317×10^{-6}	7.41
0	None	*Staphylococcus aureus*	ucld/skyline	0.9	0.6	1.48×10^{-6}	2.43158×10^{-6}	9.57
8	RGBMST	*Enterococcus faecium*	ucld/constant	0.9	0.6	6.00×10^{-7}	3.42×10^{-6}	18
8	RGBMST	*Enterococcus faecium*	ucld/skyline	0.9	0.5	-1.91×10^{-7}	9.35×10^{-6}	10
0	None	*Mycobacterium tuberculosis*	ucld/constant	1	0.9	6.26×10^{-8}	5.39148×10^{-8}	895
